# Vitamin D and Immune Function

**DOI:** 10.3390/nu5072502

**Published:** 2013-07-05

**Authors:** Barbara Prietl, Gerlies Treiber, Thomas R. Pieber, Karin Amrein

**Affiliations:** Division of Endocrinology and Metabolism, Department of Internal Medicine, Medical University of Graz, Auenbruggerplatz 15, A 8036 Graz, Austria; E-Mails: barbara.prietl@medunigraz.at (B.P.); gerlies.treiber@medunigraz.at (G.T.); endo@medunigraz.at (T.R.P.)

**Keywords:** vitamin D, autoimmunity, immune cells, adaptive immunity, innate immunity, cholecalciferol, calcitriol, 25(OH)D

## Abstract

Vitamin D metabolizing enzymes and vitamin D receptors are present in many cell types including various immune cells such as antigen-presenting-cells, T cells, B cells and monocytes. *In vitro* data show that, in addition to modulating innate immune cells, vitamin D also promotes a more tolerogenic immunological status. *In vivo* data from animals and from human vitamin D supplementation studies have shown beneficial effects of vitamin D on immune function, in particular in the context of autoimmunity. In this review, currently available data are summarized to give an overview of the effects of vitamin D on the immune system in general and on the regulation of inflammatory responses, as well as regulatory mechanisms connected to autoimmune diseases particularly in type 1 diabetes mellitus.

## 1. Introduction

The classical, hormonal actions of vitamin D are related to mineral metabolism and skeletal health. Vitamin D enhances intestinal calcium and phosphate absorption, stimulates osteoclast differentiation and calcium reabsorption from bone and promotes mineralization of the bone matrix. First evidence for the positive effect of vitamin D intake for human health came from early studies on rickets and osteomalacia (reviewed in [1]). This most severe form of vitamin D deficiency results in severe skeletal mineralization defects and frank hypocalcemia [2] and typically affects patients with serum vitamin D levels below 20 nmol/L (8 ng/mL). The historical strategy of supplementing infants with at least 200 IU (5 μg) vitamin D per day successfully decreased the incidence of rickets in the United States of America and other countries substantially, however today rickets is still not uncommon [3,4,5,6]. Vitamin D deficiency is also associated with the development of cardiovascular diseases, various types of cancer and autoimmune disorders, such as type 1 diabetes mellitus (T1D), multiple sclerosis (MS) and inflammatory bowel disease [7].

Over the last decade, the perspective on how vitamin D influences human health has changed dramatically based on the finding that the vitamin D receptor (VDR) and the vitamin D activating enzyme 1-α-hydroxylase (CYP27B1) are expressed in many cell types which are not involved in bone and mineral metabolism, such as intestine, pancreas, prostate and cells of the immune system [7,8]. This suggests an important impact of vitamin D on a much wider aspect of human health than previously known. Especially in the field of human immunology, the extra-renal synthesis of the active metabolite calcitriol—1,25(OH)_2_D—by immune cells and peripheral tissues has been proposed to have immunomodulatory properties similar to locally active cytokines [9,10]. This review provides a general summary of vitamin D and its effects on the innate and adaptive immune system. A special focus is given to autoimmune diseases and in particular to recent advances in T1D and MS.

## 2. Vitamin D and Immune Function

### 2.1. Vitamin D Sources

Vitamin D may come from three potential sources: nutritional sources, UVB-dependent endogenous production and supplements. In humans, vitamin D is mainly synthesized in the skin after exposure to UVB whereas only a minor part is derived from dietary sources. Very few natural, non-fortified products such as fatty fish (salmon, mackerel, sardines, cod liver oil) or some types of mushrooms (Shiitake), especially if sundried, contain relevant amounts of one of the two major forms, cholecalciferol (vitamin D_3_) or ergocalciferol (vitamin D_2_) [7,11,12]. Some countries like the United States and Canada fortify staple products such as dairy products with vitamin D. Thus, the individual vitamin D dietary intake is highly dependent on nutritional habits, and the country’s fortification strategy. However, a review with a global perspective found that 6 to 47% of vitamin D intake may come from dietary supplements [13,14]. Consequently, without supplementation, vitamin D status strongly depends on endogenous vitamin D production which is also influenced by genetic determinants, latitude, season, skin pigmentation and lifestyle such as the use of sunscreen and clothing [7,15].

#### Seasonality of Vitamin D Status

Because vitamin D levels have been shown to depend on season [16,17,18,19], this factor should be taken into account when interpreting an individual’s vitamin D status. Individual 25(OH)D levels reach their lowest levels after winter and their maximum at the end of summer. Interestingly, this seasonal variation resembles the described seasonal variation of some infectious diseases including sepsis [20,21].

### 2.2. Vitamin D Metabolism

In the human skin, cholecalciferol is synthesized from 7-dihydrocholesterol when exposed to UVB. Cholecalciferol is biologically inactive and immediately binds to vitamin D binding proteins or albumin. It then enters the circulation and is hydroxylated in the liver, catalyzed by the enzymes CYP2R1 and CYP27A1, which results in the production of the inactive form 25-hydroxyvitamin D—25(OH)D—which represents the main circulating vitamin D metabolite and is the most reliable parameter to define human vitamin D status [22]. In the kidney, 25(OH)D is further converted to the circulating biologically active compound calcitriol (1,25(OH)_2_D) by the enzyme 1-α-hydroxylase (CYP27B1) which is under strict control of parathyroid hormone and the phosphaturic hormone fibroblast growth factor 23 (FGF-23). Calcitriol levels are tightly regulated in a renal negative feedback loop, including inhibition of CYP27B1 by high levels of calcitriol and fibroblast growth factor 23 and stimulation of the enzyme CYP24A1 (24-hydroxylase) which metabolizes calcitriol into the inactive, water soluble form, calcitroic acid, which is then excreted in the bile. Circulating levels of calcitriol are mainly determined by renal CYP27B1 activity. However, other cell types including immune cells, also express CYP27B1 and are able to convert the inactive, circulating form 25(OH)D into the active hormone in an autocrine or paracrine manner. Especially in immune cells, such as macrophages and dendritic cells, a lack of feedback mechanisms compared to kidney cells allows the production of high local concentrations of calcitriol needed for immunomodulation (reviewed in [23]).

### 2.3. Definition of Vitamin D Status

Serum 25(OH)D is considered the most accurate marker for vitamin D status [7,24]. While the Endocrine Society advocates that levels below 20 ng/mL (50 nmol/L) should define deficiency, levels ranging from 20 to 29.9 ng/mL (52–72 nmol/L) should define insufficiency and levels above 30 ng/mL (75 nmol/L) should define sufficiency, the Institute of Medicine (IOM) considers levels of >20 ng/mL to be sufficient for the majority of the general population [25,26]. This latter classification is based largely on vitamin D’s effects on bone and mineral homeostasis. The optimal 25(OH)D serum level regarding other aspects of human health is still under debate [7,25,27]. For immune-mediated diseases, experts suggest that even higher serum 25(OH)D levels may be needed to lead to positive effects [2].

### 2.4. Safety of Supplementation in Humans

Recommendations from national health authorities for optimal serum 25(OH)D levels differ in many countries [8]. Currently, no international consensus is available on the optimal level for vitamin D supplementation, in particular on the safe upper level. While the tolerable upper daily limit given by the Endocrine Society is 10,000 IU [27], the more conservative Institute of Medicine (USA) considers a supplementation of up to 4000 IU/day to be safe [25,26]. The European Food and Safety Authority currently recommends to stay below 4000 IU/day (100 μg) [28].

Administration of the highly active metabolite calcitriol is limited because of potential side effects, in particular hypercalcemia. Active vitamin D or its analogs are rarely required except in advanced chronic kidney disease and very few other indications such as hypoparathyroidism and pseudohypoparathyroidism. The most common forms of inactive vitamin D used for supplementation are cholecalciferol (vitamin D_3_) and ergocalciferol (vitamin D_2_), shown in Table 1. While in a recent meta-analysis vitamin D3 has been found to be more efficacious in improving 25(OH)D status, especially when given as loading doses [14], both forms are considered to have an excellent safety profile including a broad therapeutic window [29,30].

Although serum 25(OH)D levels >150 ng/mL may cause acute vitamin D intoxication with hypercalcemia, hypercalciuria and calcifications in different organs [7,30,31,32,33], even prolonged daily intakes of 10,000 IU cholecalciferol are considered to be safe [29] and most cases of vitamin D intoxication have been attributed to prolonged and unintended daily intakes of >40,000 IU [34].

**Table 1:** Overview of available vitamin D preparations, their characteristics, typical indication, side effects and costs. CKD (chronic kidney disease).

	typical daily dose	indication and side effects	costs
NATIVE vitamin D			
**unhydroxylated, inactive from of vitamin D_3_** cholecalciferol **calciol **	400–4000 IU (max 10,000 IU)	vitamin D deficiency, osteoporosis therapy and prevention, hypoparathyroidism, prevention of rickets [27] hypercalcemia (rare)	inexpensive
**unhydroxylated, inactive form of vitamin D_2_** ergocalciferol **vitamin D_2_**	400–4000 IU (max 10,000 IU)		inexpensive
ACTIVE vitamin D			
**hydroxylated, active form of vitamin D** **1,25(OH)_2_D** calcitriol **1,25-dihydroxyvitamin D_3_, ** **1,25-dihydroxycholecalciferol** **analog: alfacalcidol**	0.25–1.0 μg	secondary hyperparathyroidism in advanced CKD [35], hypoparathyroidism [36,37], pseudohypoparathyroidism [38], not in vitamin D deficiency hypercalcemia/hyperphosphatemia is not uncommon (dose dependent), hypercalciuria, nephrocalcinosis	expensive
other active vitamin D analogs: ** paricalcitol, doxercalciferol (vitamin D_2_ analogs)** **falecalcitriol, maxacalcitol (vitamin D_3_ analogs) **		secondary hyperparathyroidism in advanced CKD hypercalcemia may occur, but less frequent compared with “older” active analogs	very expensive

### 2.5. Vitamin D and the Innate Immune System

Early evidence that vitamin D acts as important stimulant for innate immunity came from reports about tuberculosis treatment with cod liver oil (reviewed in [39]). More current studies specify how calcitriol enhances the antimicrobial effects of macrophages and monocytes, which are important effector cells, fighting against pathogens such as *Mycobacterium tuberculosis*. Besides enhancing chemotaxis and phagocytic capabilities of innate immune cells [23], the complex of calcitriol, VDR, and retinoid X receptor directly activates the transcription of antimicrobial peptides such as defensin β2 (DEFB) and cathelicidin antimicrobial peptide (hCAP18) [40,41,42]. In detail, monocytes exposed to *M. tuberculosis* show a strong induction of the 1α-hydroxylase CYP27B1 and the vitamin D receptor after recognizing pathogens by toll-like receptors, leading to a direct modulation of gene expression, favoring production of cathelicidin [43]. Besides toll-like receptor signaling, other cytokines such as interferon-γ or IL-4 have been found to also effect CYP27B1 expression [44]. Human cathelicidin (hCAP18) which is cleaved from LL-37 (37-residue active cationic peptide) and then causes destabilization of microbial membranes, is up-regulated in response to infections in humans and acts against bacteria, viruses and fungi [45].

In severe infections, the percentage of innate granulocytic cells such as neutrophils is very high. Early reports suggested neutrophils as main source of cathelicidin [46] but this finding is now contrasted by the report that although neutrophilic granulocytes express the VDR, they seem to have no CYP27B1 activity that would enable them to convert 25(OH)D into the bioactive form necessary to initiate cathelicidin gene expression [2]. However, in a cross-sectional analysis, serum 25(OH)D levels were found to be significantly lower in critically ill septic patients. This was associated with decreased concentrations of the antimicrobial protein cathelicidin [47]. This finding supports the theory that the vitamin D status regulates antimicrobial protein levels and may be crucial in infection control.

Low calcitriol concentrations have also been linked to elevated mortality caused by severe infections in end-stage renal disease patients [48], and low serum 25(OH)D levels have been associated with upper respiratory tract infections (URTI) [49,50,51], including influenza [52], chronic obstructive pulmonary disease [53,54] and allergic asthma [55,56]. A recent RCT (randomized controlled trial) was unable to demonstrate a reduction of URTIs with a monthly dose of 100,000 IU vitamin D3 in 322 healthy adults [57]. The major limitation of this trial certainly was the study population that was practically vitamin D replete at baseline (mean 25(OH)D 29 ng/mL). In contrast, a RCT using fortified milk in 247 severely vitamin D deficient Mongolian children (baseline 25(OH)D level 7 ng/mL led to a significant reduction of acute respiratory tract infections over the 3-month study period [58]. In a Swedish RCT in 140 immunodeficient patients, daily intake of 4000 IU cholecalciferol over one year significantly reduced infectious symptoms, the total number of specific pathogens in the nasal fluid and the use of antibiotics in the vitamin D compared to the placebo group [59].

Besides fighting directly against microbes, monocytes and other innate antigen presenting cells (APC), in particular dendritic cells (DC), are important targets for the immune modulatory effects of vitamin D. APC are responsible for the initiation of the adaptive immune response as they present antigens to T cells and B cells and are able to modulate them by either immunogenic or tolerogenic signals such as cytokines and expression of co-stimulatory molecules [60,61,62]. Different studies have shown that calcitriol and its analogs can alter function and morphology of DC to induce a more tolerogenic, immature state [23,63,64,65,66]. Immature DC are characterized by decreased levels of MHC class II and co-stimulatory molecule expression (CD40, CD80, CD86), which leads to reduced antigen presentation accompanied by a lower IL12 secretion but an increased production of the tolerogenic interleukin IL10. Calcitriol has also been described to inhibit T cell cytokines such as IL2 and IL17 and toll like receptors on monocytes [23,62]. High-dose calcitriol supplementation in healthy humans (1 μg twice daily for 7 days) leads to a significant reduction of the proinflammatory cytokine IL6 produced by peripheral mononuclear cells [67]. It is likely that a combination of all these effects results in the induction of potential regulatory T cells which are crucial for controlling immune responses and the development of autoreactivity [68].

Active and native vitamin D, calcitriol and cholecalciferol, are able to induce tolerogenic properties in DC because these cells also express the enzyme CYP27B1. This expression allows them to achieve a high local concentration of the active form of vitamin D required for immunomodulatory effects [23]. *In vitro* data are also supported by results from VDR and CYP27B1 knockout mice which show significantly increased numbers of mature DC and abnormal DC chemotaxis [69,70,71]. A recent clinical trial in 95 patients treated with adjunctive high-dose vitamin D or placebo in addition to standard tuberculosis therapy demonstrated accelerated resolution of inflammatory responses [72].

### 2.6. Vitamin D and the Adaptive Immune System

Early studies investigating the effects of vitamin D on human adaptive immune cells demonstrated an expression of the nuclear VDR as well as vitamin D-activating enzymes in both T- and B cells [73]. Notably, VDR expression by these cells is very low in resting conditions but upon activation and proliferation, T- and B cells up-regulate VDR expression significantly, allowing regulation of up to 500 vitamin D responsive genes which influence differentiation and proliferation of these cells [74,75,76].

In B cells, antiproliferative effects of calcitriol such as inhibition of differentiation, proliferation, initiation of apoptosis and decreased immunoglobulin production were initially considered to be exclusively indirectly mediated by T helper (Th) cells [74]. More recent studies confirmed additional direct effects of calcitriol on B cell homoeostasis, including inhibition of memory- and plasma-cell generation, as well as promotion of apoptosis of immunoglobulin-producing B cells [23,63,75]. This control on B cell activation and proliferation may be clinically important in autoimmune diseases as B-cells producing autoreactive antibodies play a major role in the pathophysiology of autoimmunity.

**Figure 1:**
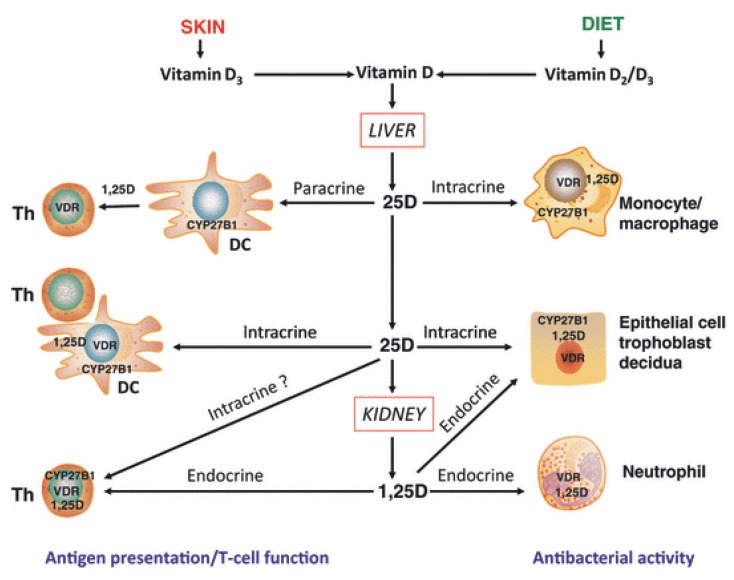
Mechanisms for innate and adaptive immune responses to vitamin D. Cholecalciferol (vitamin D3) or ergocalciferol (vitamin D2) are metabolized in the liver to form 25-hydroxyvitamin D (25D), the main circulating form of vitamin D. Target cells such as monocytes/macrophages and dendritic cells (DC) expressing the vitamin D-activating enzyme CYP27B1 and the vitamin D receptor (VDR) can then utilize 25D for intracrine responses via localized conversion to calcitriol (1,25D). In monocytes/macrophages this promotes antibacterial response to infection. In DCs, intracrine synthesis of 1,25D inhibits DC maturation, thereby modulating helper T-cell (Th) function. Th responses to 25D may also be mediated in a paracrine fashion, with DC-generated 1,25D. Intracrine immune effects of 25D also occur in CYP27B1/VDR-expressing epithelial cells. However, other cells such as neutrophils do not appear to express CYP27B1 and are therefore likely to be affected by circulating levels of 1,25D synthesized by the kidneys. VDR-expressing Th are also potential targets for systemic 1,25D, although intracrine mechanisms have also been proposed. In a similar fashion, epithelial cells, trophoblasts and decidual cells are all able to respond in an intracrine fashion to 25D, but may also respond to systemic 1,25D to promote antibacterial responses. With permission from Clinical Endocrinology [2].

The other major type of adaptive immune cells, T cells, is also thought to be an important target for the immunomodulatory effects of different forms of vitamin D. In a recent review [2] four potential mechanisms by which vitamin D may influence T cell function have been proposed (Figure 1):

1. direct, endocrine effects on T cells mediated via systemic calcitriol.
2. direct, intracrine conversion of 25(OH)D to calcitriol by T cells.
3. direct, paracrine effects of calcitriol on T cells following conversion of 25(OH)D to calcitriol by monocytes or dendritic cells.
4. indirect effects on antigen presentation to T cells mediated via localized APC affected by calcitriol.

In principle, vitamin D exposure leads to a shift from a proinflammatory to a more tolerogenic immune status, including very diverse effects on T cell subtypes: Calcitriol suppresses T helper (Th) cell proliferation, differentiation and modulates their cytokine production [77]. In particular, treatment of T cells with calcitriol or analogs inhibits the secretion of proinflammatory Th1 (IL2, interferon-γ, tumor necrosis factor α), Th9 (IL9) and Th22 (IL22) cytokines [78,79,80,81,82,83], but promotes the production of more anti-inflammatory Th2 cytokines (IL3, IL4, IL5, IL10) [84]. IL17 producing Th17 cells are also affected by vitamin D. Inhibition of Th17 activity seems to play a major role in the treatment of autoimmune diseases as shown in non-obese diabetic (NOD) mice [85]. Recently, calcitriol was found to directly suppress IL17 production on a transcriptional level [86] and activated human T-cells exposed to calcitriol produced significantly decreased levels of IL17, interferon-γ and IL21 [87]. The same study also revealed a change towards a tolerogenic phenotype, including increased expression of genes typical for regulatory T cells (Tregs), by adding a combination of calcitriol and IL2 to human primary T cell cultures.

Tregs act to suppress proinflammatory responses by other immune cells and aim to prevent exaggerated or autoimmune responses [88]. They are potently induced by different forms of vitamin D [89]. Tregs can be induced and stimulated by vitamin D in an indirect pathway, via antigen-presenting cells (APC), including the group of dendritic cells (DC) which stay in an immature state upon vitamin D treatment and therefore present less antigens. The direct pathway acts via systemic calcitriol effects or intracrine conversion of 25(OH)D to calcitriol by Tregs themselves. Administration of calcitriol to renal transplant recipients expanded the circulating Treg population [90]. To date, studies of vitamin D and T cell function have primarily focused on the response of these cells to active calcitriol or analogs [79,82,87]. There is a lack of studies investigating the effects of native vitamin D forms (ergo-/cholecalciferol) supplementation on the activity of different T cell subtypes. In our own pilot study and the subsequent randomized, placebo controlled trial in healthy subjects, the percentage of Tregs in peripheral blood increased significantly after supplementation with high doses of cholecalciferol [91,92]. Using cholecalciferol supplementation as adjunctive therapy in new onset T1D patients, the percentage of peripheral Tregs increased, although there was no significant difference in %Tregs between placebo and treatment group after one year of supplementation [93].

Taken together these results suggest that vitamin D may not only support the innate but also the adaptive immune system. Vitamin D supplementation could also provide a safe and useful future therapy to support immune tolerance in autoimmune diseases or following transplantation [94].

### 2.7. Vitamin D and Autoimmune Diseases

Autoimmune diseases are characterized by a loss of immune homeostasis resulting in corrupted self-antigen recognition followed by the destruction of body tissue by autoreactive immune cells. A combination of genetic predisposition [95], epidemiological risk factors [96] and environmental contributors contributes to the development of autoimmune diseases. One important factor may be the availability of sufficient vitamin D levels [97,98] as various epidemiological studies suggest associations between vitamin D deficiency and a higher incidence of autoimmune diseases, such as T1D, MS, systemic lupus erythematosus (SLE), rheumatoid arthritis (RA) and inflammatory bowel disease (IBD).

In animal models for T1D, MS, SLE, IBD and autoimmune uveitis, administration of calcitriol either prevented or ameliorated autoimmunity. Studies with vitamin D deficient or VDR knock-out animals show increased inflammation and susceptibility to T1D and Crohn’s disease, disturbed T cell homing and lack of host protection from bacterial invasion and infection (reviewed in [99]).

Over the last 40 years, several clinical studies addressed the questions whether vitamin D levels in humans are associated with the risk of developing autoimmunity and whether development and progression of autoimmune diseases can be influenced by vitamin D supplementation. A recent systematic review analyzed results from 219 published studies and concluded that vitamin D seems to play a beneficial role in the prevention of autoimmunity but that there is still a lack of randomized controlled clinical trials in this field [100]. In the following section we have summarized the most relevant studies associating vitamin D insufficiency and autoimmunity in the two best studied autoimmune diseases T1D and MS:

#### 2.7.1. Type 1 Diabetes Mellitus

The chronic autoimmune disease T1D usually results from a T cell mediated destruction of insulin producing, pancreatic β-cells with a typical onset in childhood or adolescence. The worldwide incidence rate of T1D is steadily increasing and accumulating data show that it is correlated with an insufficient vitamin D [101,102,103]. On the other hand, there is evidence that vitamin D supplementation early in life is a protective factor against the development of T1D (reviewed in [104,105]). For example, a substudy of EURODIAB revealed a 33% reduced risk of developing T1D for children who received vitamin D supplementation during their first year of life [106]. A meta-analysis of four large studies also supported these results and showed a significantly reduced risk (pooled odds ratio 0.71) in infants to develop T1D when receiving vitamin D supplementation [105]. Furthermore, in animal models, such as the NOD mice, the administration of calcitriol or vitamin D analogs prevented or at least delayed the onset of diabetes [107,108,109].

Controlled trials with active and inactive vitamin D have been conducted in new-onset T1D and have shown controversial results (Table 2). Two studies of calcitriol supplementation in T1D patients did not show a positive effect on residual β-cell function [110,111]. Recently however, loss of β-cell function was attenuated in a randomized, double-blind, placebo-controlled clinical trial using 2000 IU of cholecalciferol for 18 months in 38 T1D patients [93].

To adequately study the prevention of autoimmune onset by supplementing vitamin D in a randomized controlled trial, a large number of children who are genetically at risk would have to be recruited. Susceptibility to T1D has been found to be associated with variation in the CYP27B1 gene [112]. To date, many interventional trials on vitamin D and T1D are currently ongoing that will hopefully extend our knowledge on this topic greatly in the near future.

**Table 2:** Randomized controlled trials on vitamin D treatment in type 1 diabetes (T1D) and latent autoimmunity diabetes in adults (LADA).

author, year, country [reference]	sample size included (completed)	Subjects	Intervention (type, dose, duration)	Study results
Gabbay 2012, Brazil [93]	38 (35)	new onset T1D (≤6 months) fasting C-peptide ≥ 0.6 ng/mL age 7–30 years	cholecalciferol (vitamin D3) oral, 2000 IU daily 18 months	=insulin needs, HbA1c slower decline of residual beta -cell function, protective immunologic effect including higher number of regulatory T-cells
Bizzarri 2010, Italy [111]	34 (27)	new onset T1D < 12 weeks basal C-peptide 0.25 nmol/L age 11–35 years	calcitriol (1,25(OH)2D3) oral, 0.25 μg/day 2 years	=insulin needs =C-peptide levels =HbA1c
Walter 2010, Germany [110]	40 (38)	new onset T1D < 2 months age 18–39 years	calcitriol (1,25(OH)2D3) oral, 0.25 μg/day 9 months	=insulin needs =C-peptide levels =HbA1c
Li 2009, China [113]	35 (35)	LADA, diagnosis < 5 years age > 20 years	alphacalcidol 1α(OH)D3 oral, 0.25 μg 2×/day 1 year	slower decline of residual beta-cell function (diagnosis < 1 year)
Pitocco 2006, Italy [114]	70 (67)	new onset T1D < 4 weeks age > 5 years	calcitriol (1,25(OH)2D3) oral, 0.25 μg on alternate days 1 year	↓ insulin needs (at 3 and 6 months only) =C-peptide levels =HbA1c

Symbols: ≤ less than or equal to; ≥ greater than or equal to; < less than; > greater than; = not affected; ↓ decreased.

#### 2.7.2. Multiple Sclerosis

Hypovitaminosis D is associated with an increased risk to develop multiple sclerosis [115,116]: an autoimmune disease characterized by T cell mediated inflammation in the cerebral nervous system. Vitamin D supplementation in humans resulted in a reduced risk of developing MS [117] and the use of vitamin D as an add-on therapy in combination with interferon-β reduced disease activity [118]. Contrasting this, recent placebo controlled, randomized trials revealed no beneficial effects of high dose vitamin D supplementation on MRI brain lesions in relapsing-remitting MS [119] and no improvement in the annualized relapse rate, expanded disability status or MS-functional composite [120]. In the murine model for multiple sclerosis (experimental autoimmune encephalomyelitis, EAE), oral administration of calcitriol prevents disease onset and modulates T cell composition towards more anti-inflammatory status, such as decreasing the number of Th17 cells in the central nervous system [121]. In humans, a correlation between serum 25(OH)D levels and Treg activity has been reported [122,123].

Recently, Smolders *et al.* [124] suggested an endogenous role for vitamin D in the suppression of active MS lesions based on increased expression of VDR in the normal appearing white matter of MS patients as well as elevated expression of VDR and CYP27B1 in chronic active MS lesions compared to tissue from healthy controls. Several interventional trials on vitamin D and MS using relatively high doses of native vitamin D such as the *SOLAR* or the *EVIDIMS* study are currently ongoing and promise to elucidate many aspects in the coming years.

## 3. Conclusion and Future Perspectives

In recent decades, vitamin D research has confirmed important interactions between vitamin D and cells from the innate as well as from the adaptive immune system. Data have shown that a broad spectrum of tissue cells, including immune cells, express vitamin D metabolizing enzymes, providing a biologically plausible mechanism for local, auto- and paracrine conversion of the native circulating forms, to the active form calcitriol. This process seems to be essential for normal immune function and therefore impaired or insufficient vitamin D levels may lead to dysregulation of immune responses. Addressing the questions as to whether vitamin D levels are related to the risk of developing autoimmunity and whether vitamin D supplementation can modify the course of autoimmune diseases, a recent systematic review [100] reached the conclusion that several studies performed over the last 40 years support the role of vitamin D in the prevention of autoimmune diseases but that there is still a lack of randomized controlled clinical trials in this field.

So far, there is no worldwide consensus about the recommended targeted serum level and the optimal mode of vitamin D supplementation, including the question whether different forms of vitamin D, such as vitamin D_2_, vitamin D_3_ or vitamin D analogs have particular advantages for variable immunomodulatory responses. In the future, more and larger clinical trials are needed to determine how vitamin D supplementation affects the pathophysiology of different diseases *in vivo* and how it may contribute to better efficacy of conventional therapies by immunomodulation. Questions about the optimal mode and dosage of supplementation also have to be answered in these future trials. However, taking all current evidence together, vitamin D emerges as a promising and relatively safe nutrient for new strategies in the prevention and adjunctive treatment of diseases caused by impaired immune-homeostasis.
